# Development of an m6A-Related lncRNAs Signature Predicts Tumor Stemness and Prognosis for Low-Grade Glioma Patients

**DOI:** 10.1155/2024/2062283

**Published:** 2024-01-09

**Authors:** Dahua Xu, Peihu Li, Chunrui Zhang, Yutong Shen, Jiale Cai, Qingchen Wei, Meng Cao, Zhizhou Xu, Deng Wu, Hong Wang, Xiaoman Bi, Bo Wang, Kongning Li

**Affiliations:** ^1^Key Laboratory of Tropical Translational Medicine of Ministry of Education, College of Biomedical Information and Engineering, Hainan General Hospital, Hainan Affiliated Hospital of Hainan Medical University, Haikou 571199, China; ^2^Institute of Genetics and Developmental Biology, Chinese Academy of Sciences, Beijing 100020, China; ^3^School of Life Sciences, Faculty of Science, The Chinese University of Hong Kong, Hong Kong 999077, China

## Abstract

**Background:**

Growing evidence has revealed that m6A modification of long noncoding RNAs (lncRNAs) dynamically controls tumor stemness and tumorigenesis-related processes. However, the prognostic significance of m6A-related lncRNAs and their associations with stemness in low-grade glioma (LGG) remain to be clarified.

**Methods:**

A multicenter transcriptome analysis of lncRNA expression in 1,247 LGG samples was performed in this study. The stemness landscape of LGG tumors was presented and associations with clinical features were revealed. The m6A-related lncRNAs were identified between stemness groups and were further prioritized via least absolute shrinkage and selection operator Cox regression analysis. A risk score model based on m6A-related lncRNAs was constructed and validated in external LGG datasets.

**Results:**

Based on the expression of LINC02984, PFKP-DT, and CRNDE, a risk model and nomogram were constructed; they successfully predicted the survival of patients and were extended to external datasets. Significant correlations were observed between the risk score and tumor stemness. Moreover, patients in different risk groups exhibited distinct tumor immune microenvironments and immune signatures. We finally provided several potential compounds suitable for specific risk groups, which may aid in LGG treatment.

**Conclusions:**

This novel signature presents noteworthy value in the prediction of prognosis and stemness status for LGG patients and will foster future research on the development of clinical regimens.

## 1. Introduction

Low-grade glioma (LGG) refers grades II and III gliomas, as defined by The Cancer Genome Atlas (TCGA) classification [1]. A large majority of LGG patients have isocitrate dehydrogenase (IDH) mutations, and LGG patients have better survival outcomes than glioblastoma patients. However, most LGG patients eventually develop glioblastoma (GBM), which is resistant to chemotherapy and radiotherapy, thus resulting in high mortality [2]. Moreover, novel independent factors for survival risk stratification of LGG patients need to be identified, as the prognoses are within a similar range for those grades II and III patients with IDH mutation [3]. Cancer stem cells (CSCs) are a class of pluripotent cells with the capabilities of self-renewal, unrestricted proliferation, and multidirectional differentiation [4]. Stemness refers to the differentiation potential of CSCs. Stemness has been proven to play important roles in glioma progression and treatment resistance [5]. Deep sequencing and computational approaches are well established to provide insight for the estimation of cancer stemness, such as the gene expression-based stemness index (mRNAsi) developed by a one-class logistic regression (OCLR) machine learning algorithm [6]. Therefore, predictive regimens combining these stemness indices are imperative for LGG patients.

N6-methyladenosine (m6A) is the most abundant modification in mammalian RNA molecules. It affects RNA stability, translation, translocation, and splicing [7]. m6A-binding proteins (readers), demethylases (erasers), and methyltransferases (writers) constitute the m6A regulation loop [8]. Evidence has highlighted the roles of m6A in CSC generation and maintenance and in turn its influence on the carcinogenic process and therapeutic resistance [9]. Dixit et al. [10] found that the m6A reader YTHDF2 could stabilize MYC mRNA in glioma stem cells, which promotes oncogenic pathway activation and tumor growth. Long noncoding RNAs (lncRNAs) have been found to play important roles in glioma cancers and can be modified by m6A modification. The stability of MALAT1 is mediated by the m6A writer METTL3 and further activates NF-*κ*B signaling to promote IDH-wild-type glioma progression [11]. The m6A reader IGF2BP3 was found to regulate WEE2-AS1, which further promotes glioblastoma progression through the stabilization of the RPN2 protein and activation of the downstream PI3K-Akt signaling pathway [12]. However, the efficiency of m6A-related lncRNAs in reflecting cancer stemness heterogeneity and their ability to stratify LGG patients into different prognostic risk groups are still largely unknown.

In this study, the stemness index was first evaluated based on multicenter transcriptome data for LGG patients. The m6A-related lncRNAs were identified, and potential functions were inferred in different stemness groups. Then, an m6A-related lncRNA prognostic signature was constructed based on the predictive capacity of LINC02984, PFKP-DT, and CRNDE. Risk groups with distinct overall survival outcomes and stemness scores were categorized into training and validation datasets. The differences in the tumor immune microenvironment (TIME) and immune signatures were compared between risk groups. CMap analysis identified several potential compounds ideal for treating different risk groups. The risk model that we presented will improve the accuracy of prognosis prediction for LGG patients.

## 2. Materials and Methods

### 2.1. LGG Dataset Collection and Processing

The RNA-sequencing profiles for lower grade gliomas were collected from TCGA (https://portal.gdc.cancer.gov/) and the Chinese Glioma Genome Atlas (CGGA, http://www.cgga.org.cn/) datasets [13]. Only primary tumor samples with WHO grades II and III were included in this study. This yielded 511 samples from the TCGA-LGG cohort, 282 samples from the CGGA-693, and 144 samples from the CGGA-325 cohort. Gene expression levels were quantified by fragments per kilobase million values. These LGG datasets provided sample-matched clinical information, which included overall survival time, vital status, age, gender, WHO grade, IDH mutation, 1p/19q codeletion, and O6-methylguanine-DNA methyltransferase (MGMT) promoter methylation. We also obtained two microarray datasets of LGG for the Affymetrix Human Genome U133 Plus 2.0 Array platform as external datasets, which included 162 patients from GSE108474 and 148 patients from E-MATB-3892. The annotation of lncRNAs was downloaded from GENCODE V34. Only lncRNAs presented in TCGA and CGGA were retained for further analysis. All expression profiles were log2 transformed. The characteristics of the datasets used in this study are shown in Table S1.

### 2.2. Stemness Estimation for LGG Samples

The gene expression-based stemness index (mRNAsi) was calculated via the OCLR algorithm [6], which ranged from 0 to 1. The median of the mRNAsi score was set as the cutoff to define the mRNAsi-high and mRNAsi-low groups. Twenty-nine stem cell gene sets from the Molecular Signature Database (MsigDB, v2022.1.Hs), and 26 gene sets from StemChecker (Table S2) were also collected. StemChecker is a systematic web server that collects stemness signatures through the curation of transcriptome analysis, RNAi screens, transcription factor target gene sets, literature retrieval, and computational derivation [14]. The single-sample GSEA (ssGSEA) algorithm was applied to estimate the enrichment of stemness gene sets for LGG patients [15].

### 2.3. Identification of m6A-Related lncRNAs and Construction of the ceRNA Network

A gene set of 23 m6A regulators, including 13 readers (LRPPRC, RBMX, HNRNPA2B1, HNRNPC, FMR1, YTHDF3, YTHDF1, YTHDF2, IGF2BP2, IGF2BP3, IGF2BP1, YTHDC1, and YTHDC2), eight writers (ZC3H13, METTL3, RBM15, RBM15B, VIRMA, WTAP, METTL16, and METTL14), and two erasers (FTO and ALKBH5), was obtained from the literature. The associations between lncRNAs and m6A regulators were calculated by Spearman's rank correlation analysis. Only lncRNAs with an absolute Spearman correlation coefficient (*ρ*) > 0.3 and *p*-adjusted < 0.05 (FDR method) were considered m6A-related lncRNAs. We further assessed the lncRNA-mediated ceRNA network in LGG patients via the Lnc2m6A webserver (http://hainmu-biobigdata.com/Lnc2m6A), which revealed ceRNA interactions by integrating experimental miRNA regulatory information and coexpression analysis [16].

### 2.4. Development and Validation of the m6A-Related lncRNA Risk Score Model

Univariate Cox regression was first performed to identify prognostic m6A-related lncRNAs in the TCGA-LGG cohort. Then, least absolute shrinkage and selection operator (LASSO) Cox regression was used to filter essential lncRNAs via the glmnet R package. We used 10-time cross-validation to assess the optimal values for *λ* by the lambda.1se function. The predictive model was established as previously described [17, 18], and a multivariable Cox regression model was constructed based on the selected lncRNA features in the training dataset. A risk score model was then proposed as follows:

(1) $$\begin{array}{c} \text{Risk score}\left(\text{RS}\right)=\sum_{i=1}^{N}\left({\text{Coe}}_{i}\times {\text{Exp}}_{i}\right), \end{array}$$

where *N* is the number of m6A-related lncRNAs after filtration, Coe_*i*_ is the regression coefficient of lncRNA_*i*_ obtained from the multivariable Cox regression analysis, and Exp_*i*_ is the expression level of lncRNA_*i*_ in LGG patients.

The regression coefficient and the cutoff value (median risk score) in the training dataset were retained and applied to the CGGA-693 and CGGA-325 cohorts to calculate the risk score. LGG patients in each dataset were divided into high- and low-risk groups based on the same cutoff. Due to the differences between sequencing and array platforms, the regression coefficients from the training dataset were used for the GSE108474 and E-MATB-3892 datasets, and the median risk score in each dataset was used as the cutoff. The difference in overall survival between the two risk groups was assessed by the log-rank test. Time-dependent receiver operating characteristic (ROC) curves were utilized to estimate the sensitivity and specificity of the m6A-related lncRNA risk model for LGG patients.

### 2.5. Nomogram Analysis

The nomogram analysis was performed following the methods of Guo et al. [19]. Clinical features, including age, sex, grade, IDH mutation, 1p/19q codeletion, MGMT methylation, and our risk score, were included in the multivariable Cox regression model in the TCGA-LGG training dataset. Variables with significant prognostic efficacy were used to create a nomogram for LGG patients via the rms R package. Based on the constructed nomogram, the formula for prognostic features and clinical-related points was obtained from the nomogramEx R package and then applied to the CGGA-693 and CGGA-325 cohorts to calculate nomogram points. Time-dependent ROC and decision curve analyses were also performed to evaluate the applicability of the nomogram.

### 2.6. Immune Infiltration and Immune Signature Estimation

To estimate the immune cell infiltration for LGG patients, we assessed the TCGA-LGG, CGGA-693, and CGGA-325 expression profiles via the TIMER 2.0 (http://timer.cistrome.org/) website [20] and obtained the predicted cell infiltration levels for 22 immune cell types based on the leucocyte signature matrix 22 (LM22) signatures using the CIBERSORT algorithm [21]. The immune signature, including immune checkpoints, cytolytic activity (CYT), human leukocyte antigen (HLA), interferon (IFN) response, and tumor-infiltrating lymphocytes (TILS), was corrected from a previous study [22]. We also collected 67 immune-regulatory genes, which included 45 immune stimulators and 22 immune inhibitors from Vidotto et al.'s [23] study. ssGSEA was applied to estimate the activities of immune signatures in LGG patients.

### 2.7. Connectivity Map (CMap) Analysis

CMap (https://clue.io/) was used to predict potential compounds associated with different risk groups of LGG patients. The top 150 differentially expressed genes were first imported to the tool, and only compounds with exact targets and mode of action (MoA) data were retained. The remaining compounds with a negative enrichment score and FDR < 0.05 were considered potential therapeutic drugs.

### 2.8. Statistical Analysis

The statistical analyses in this study were performed by R 4.1.1 software. The m6A-related ceRNA network was visualized by Cytoscape 3.7.2. Functional enrichment analysis of ceRNAs was performed by Metascape with *Homo sapiens* as the selected parameter. The Kolmogorov‒Smirnov test (ks.test in R software) was used to estimate the normality of the distribution for the expression level, mRNAsi index, stemness gene set score, immune cell infiltration, and immune signature data. We found that 90.60%, 99.29%, and 96.39% of lncRNAs showed non-normally distributed expression patterns, and 58.66%, 93.71%, and 84.44% of protein-coding genes expression showed non-normally distributed expression patterns in the TCGA-LGG, CGGA-693, and CGGA-325 datasets, respectively. These results were consistent with previous findings that less than 50% of protein-coding genes were normally distributed in the TCGA transcriptome [24]. Only the mRNAsi was normally distributed, while the other variables were not normally distributed. Considering the distribution of these features, we used Student's *t*-test to estimate the differences in the mRNAsi between LGG groups, and Wilcoxon's rank sum tests were used to estimate the differences in expression level, stemness gene set score, immune cell infiltration, and immune signature score. In addition, Spearman correlation was used for correlation analysis. The differences in clinical features, including sex and age, between LGG groups were identified by the chi-squared test to estimate potential bias. GSEA was implemented via the clusterProfiler R package [25]. Nonparametric Kaplan‒Meier survival curve analysis is one of the best options for survival analysis [26]. We used Kaplan‒Meier curves to estimate the survival status of LGG patients, and the significance of survival differences between LGG groups was estimated by the log-rank test. Differences with a *p* value less than 0.05 were considered significant.

## 3. Results

### 3.1. The Stemness Index Is Associated with LGG Clinical Features

**Figure 1:**
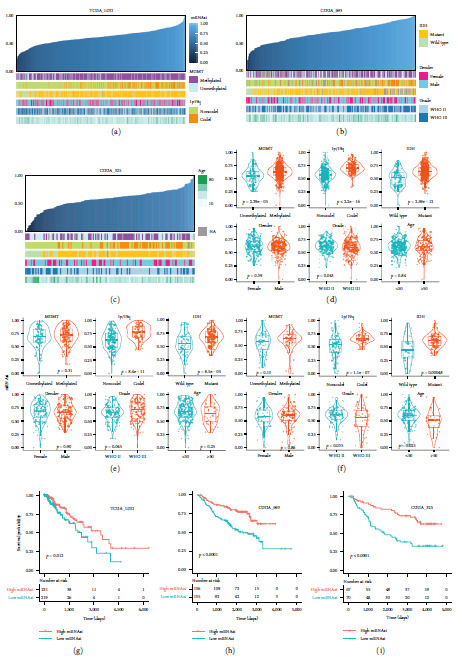
The clinical features associated with the mRNAsi index in LGG patients. (a–c) The overview of the correlation between mRNAsi and clinical features for TCGA-LGG, CGGA-693, and CGGA-325 cohorts. Columns represent LGG samples ranked by mRNAsi from low to high. (d–f) Violin plots of mRNAsi in LGG patients classified by MGMT methylation, 1p/19q codeletion, IDH mutation, gender, grade, and age for TCGA-LGG, CGGA-693, and CGGA-325 cohorts. Differential analysis between the LGG groups was estimated by the Student's *t*-test. (g–i) Kaplan–Meier survival plots for stemness-high and stemness-low patients for TCGA-LGG, CGGA-693, and CGGA-325 cohorts.

To explore the relationship between cancer stemness and clinical features, we first employed the OCLR algorithm based on the gene expression profiles of 511 TCGA-LGG patients, 282 CGGA-693 patients, and 144 CGGA-325 patients. Then, patients were ranked from low to high based on the mRNAsi, and the matched clinical features are shown in Figure 1(a)–1(c). We found that the patients with MGMT promoter methylation, 1p/19q codeletion, and IDH mutation demonstrated significantly higher mRNAsi scores (Figure 1(d)–1(f)). No significant correlation between sex and mRNAsi was observed. In addition, patients with grade III exhibited lower mRNAsi scores than those with grade II in the TCGA-LGG and CGGA-325 cohorts, while the phenomenon was reversed in the CGGA-693 dataset (Figure 1(d)–1(f)). As shown in Figure 1(f), patients aged <50 years displayed significantly higher mRNAsi scores than older patients. All these results were partly consistent with the analysis of the stemness landscape for glioblastoma patients [27]. Two common biomarkers of LGG somatic mutation provided by the TCGA center were also included. The stemness index was significantly lower in alpha-thalassemia/mental retardation, X-linked (ATRX) mutant samples than in wild-type samples but significantly higher in the telomerase reverse transcriptase (TERT) mutant group (Figure S1). Based on the median value, LGG patients were divided into mRNAsi-high and mRNAsi-low groups. K‒M survival analysis revealed that the high mRNAsi group presented significantly better survival outcomes than the mRNAsi-low group in all three LGG cohorts (Figure 1(g)–1(i)). These results indicate a strong association between the stemness index and clinical features of LGG patients.

### 3.2. Identification of m6A-Related lncRNAs in LGG Stemness Groups

**Figure 2:**
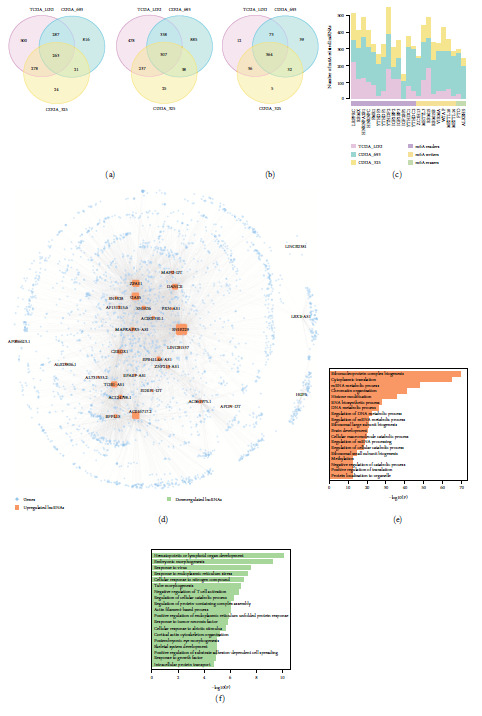
Identification and functional analysis of m6A-related lncRNAs between stemness groups. (a) Venn plot showing the overlap of upregulated and downregulated lncRNAs among TCGA-LGG, CGGA-693, and CGGA-325 cohorts. Differential analysis between the LGG groups was estimated by the Wilcoxon's rank sum tests. (b) Venn plot showing the overlap of m6A-related lncRNAs among LGG cohorts. (c) The distribution of m6A regulators for regulating lncRNAs across LGG cohorts. (d) The ceRNA network for m6A-related lncRNAs. The circle node represents protein-coding genes, the square node represents lncRNAs, and the node size reflects the note degree in the network. (e, f) The enriched biological process of genes linked with upregulated and downregulated genes in the ceRNA network.

The interplay between m6A and noncoding RNAs plays pivotal roles in modulating cancer stemness [28]. To identify potential m6A-related lncRNAs contributing to LGG stemness, the differential expression of lncRNAs between the mRNAsi-high and mRNAsi-low groups was first explored. Since no bias for sex and age was observed between LGG groups (Figure S2, chi-square test *p* > 0.05), we performed the Wilcoxon rank-sum test for differential analysis, as previously described [29]. In total, we identified 1,328, 1,387, and 586 upregulated lncRNAs as well as 1,360, 1,568, and 607 downregulated lncRNAs in the TCGA-LGG, CGGA-693, and CGGA-325 cohorts, respectively (mRNAsi-high vs. mRNAsi-low, Wilcoxon's rank sum test FDR < 0.05). There were 263 upregulated and 307 downregulated lncRNA that shared similar expression patterns across multicenter LGG datasets (Figure 2(a)). We next estimated the associations of these lncRNAs with m6A regulators by Spearman correlation analysis and identified 364 robust m6A-related lncRNAs (Figure 2(b)). These lncRNAs were significantly overlapped with the m6A-related lncRNAs in the LGG tumor type provided from Lnc2m6A, which integrated high-confidence targets for m6A regulators and computational methods (overlap number = 242, hypergeometric test *p* value < 2.2e–16). A balanced distribution was observed between lncRNAs and 23 m6A regulators (Figure 2(c)). Among them, YTHDF2 was associated with the highest number of lncRNAs, while no significant correlations were acquired for IFG2BP1 and ALKBH5 in the TCGA-LGG cohort.

To explore the potential biological function of m6A-related lncRNAs, we used the ceRNA tools in Lnc2m6A and extracted coexpressed lncRNA/gene pairs to construct a ceRNA network. The network comprised 36 m6A-related lncRNAs, 2,562 protein-coding genes, and 8,421 interactions (Figure 2(d)). Functional enrichment analysis based on the genes related to 26 upregulated and three downregulated lncRNAs in the ceRNA network was performed. The ribonucleoprotein complex biogenesis, cytoplasmic translation, brain development, and several metabolic processes were found to be related to upregulated lncRNAs (Figure 2(e)). For downregulated lncRNAs, their interacting genes were enriched in immune regulation processes, such as hematopoietic or lymphoid organ development and negative regulation of T cell activation and in cancer-related functions, such as response to tumor necrosis factor (Figure 2(f)). These results highlight the essential roles of m6A-related lncRNAs in tumorigenesis.

### 3.3. The m6A-Related lncRNA Risk Score Exhibits Prognostic Efficiency and Reveals Tumor Stemness Heterogeneity

**Figure 3:**
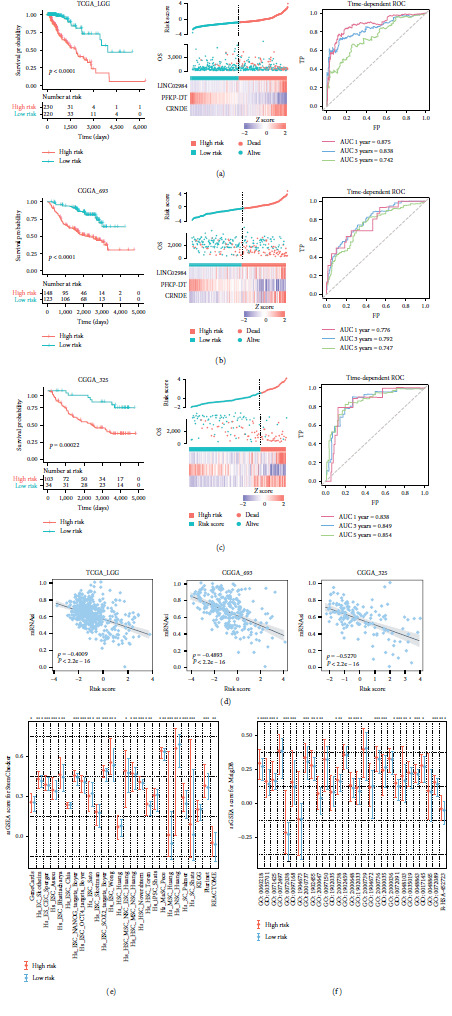
Construction of m6A-related lncRNA risk score and its correlation with tumor stemness. (a–c) The performance of risk score in TCGA-LGG, CGGA-693, and CGGA-325 cohorts. The left panel showed the Kaplan–Meier survival plot for high-risk and low-risk patients. The middle panel showed risk score distribution, patients' survival status, and heatmap of three lncRNA expression profiles. The black dotted line represents the cutoff obtained from the training dataset. The right panel showed the time-dependent ROC of the risk score for predicting 1-, 3-, and 5-year outcomes. (d) Scatter plots showing the correlation between risk score and mRNAsi index across LGG cohorts. (e, f) Box plots showing the activities of StemChecker and MsigDB stemness categories estimated by ssGSEA in LGG risk groups.  ^*∗*^*p* < 0.05,  ^*∗∗*^*p* < 0.01, and  ^*∗∗∗*^*p* < 0.001, and ns for no significant.

To develop a prognostic signature for LGG patients, univariate Cox regression was performed based on the 364 m6A-related lncRNAs expression in the TCGA-LGG training set. In total, 238 candidate lncRNAs were identified to be associated with LGG overall survival; these included 116 risk-related factors and 122 protective factors. LASSO-Cox analysis was further performed using the 238 lncRNAs mentioned above, and three m6A-related lncRNAs (LINC02984, PFKP-DT, and CRNDE) were retained according to the optimal *λ* value (Figure S3). We next calculated the risk score for all LGG datasets based on the multivariable Cox regression coefficient of three lncRNAs (LINC02984: 0.7096, PFKP-DT: −1.0337, and CRNDE: 0.5098) and split the patients into high- and low-risk groups using the cutoff (the median risk score in the training dataset). The OS of the patients in the high-risk group was worse than that of patients in the low-risk group. The patients were then ranked according to the corresponding risk scores, and the dead patients tended to be included in the high-risk group. Consistent with the coefficients, the expression levels of LINC02984 and CRNDE were elevated in the high-risk group, and the expression level of PFKP-DT was decreased in this group. Moreover, the time-dependent AUCs of the risk model in predicting 1-, 3-, and 5-year OS were 0.875, 0.838, and 0.742, respectively (Figure 3(a)). Using the same coefficient and cutoff, similar results were also observed in the CGGA-693 and CGGA-325 datasets (Figures 3(b) and 3(c)). Similarly, no bias for sex or age was observed between LGG risk groups (Figure S4, chi-square test *p* > 0.05). Moreover, LGG patients with high-risk scores exhibited poorer OS than those with low-risk scores in two external microarray datasets (Figure S5). Collectively, these results indicated the prognostic efficiency of an m6A-related lncRNA risk score for LGG survival.

The relationship between the risk score and tumor stem cells was further estimated. In general, we found that the risk score was negatively correlated with the mRNAsi in all three LGG cohorts (Figure 3(d), *p* value < 2.2e–16). The unique stemness status of brain cells leads to the restricted negative correlations between mRNAsi and tumor pathological features for LGG [6], which may introduce bias in stem cell landscape analysis. Thus, stem cell gene sets from two public resources (StemChecker and MsigDB) were also included to estimate tumor stemness. The gene set activities were globally increased in the high-risk groups for all datasets (Figures 3(e) and 3(f), Figure S6), which verified that the m6A-related risk score could reveal tumor stemness heterogeneity.

### 3.4. Nomogram Construction and Validation

**Figure 4:**
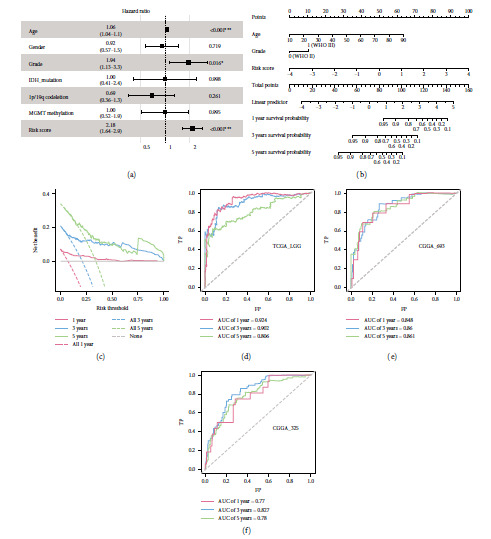
Prognostic independence evaluation and nomogram construction. (a) Forest plot for the multivariable Cox regression model in TCGA training dataset. (b) Nomogram constructed by the age, grade, and risk score for predicting 1-, 3-, and 5-year OS. (c) Decision curve of the nomogram for 1-, 3-, and 5-year OS. (d–f) Time-dependent ROC of the nomogram points for predicting 1-, 3-, and 5-year OS outcome for TCGA-LGG, CGGA-693, and CGGA-325 cohorts.

As the risk score alone may not be sufficient for predicting LGG prognosis, we followed the methods of Guo et al. [19] to construct a nomogram in combination with other clinical features. In accordance with the multivariable Cox regression model (Figure 4(a)), age, grade, and risk score were independent indicators of LGG OS. Among them, the risk score had the highest predictive power. Thus, a 1-, 3-, and 5-year OS nomogram was constructed based on these factors, and the risk score remained a good reference and predictive marker for clinicians (C-index = 0.874, Figure 4(b)). As shown in the DCA (Figure 4(c)), the nomogram had a great benefit for predicting LGG OS. According to the nomogram points, the time-dependent AUCs (0.924, 0.902, and 0.806 for 1-, 3- and 5-year survival prediction, respectively, in the TCGA training dataset) were better than those of the risk score alone (Figure 4(d)). The efficiency of the nomogram was further verified in external datasets based on the same point formula, and high performance was also observed in the CGGA-693 and CGGA-325 cohorts (Figures 4(e) and 4(f)).

### 3.5. Functional Characterization of the m6A-Related lncRNA Risk Score

**Figure 5:**
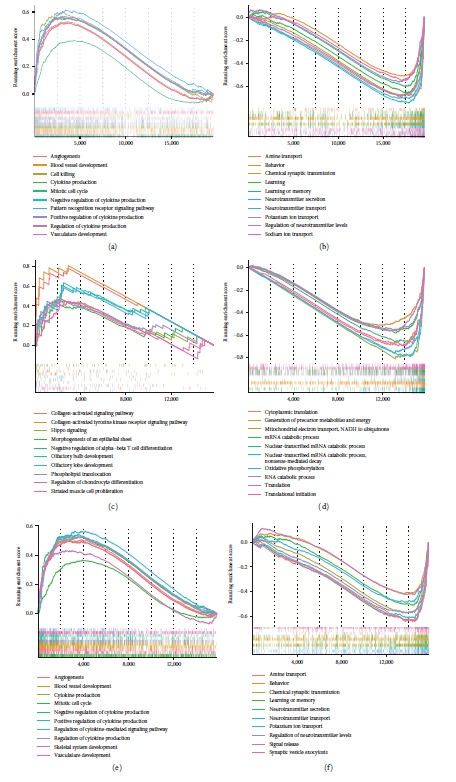
GSEA analysis for LGG risk groups. (a, b) Top 10 upregulated and downregulated enriched biological processes between high- and low-risk groups in the TCGA-LGG dataset. (c, d) Top 10 upregulated and downregulated enriched biological processes between high and low-risk groups in the CGGA-693 dataset. (e, f) Top 10 upregulated and downregulated enriched biological processes between high- and low-risk groups in the CGGA-325 dataset.

GSEA was utilized to identify potential biological processes associated with the m6A-related lncRNA risk score according to the ordered gene list of high-risk compared to low-risk groups. In the TCGA-LGG cohort, angiogenesis, blood vessel development, and several immune regulation processes were all enriched in the high-risk group (Figure 5(a)). Neurotransmitter secretion and transport, behavior, and learning or memory functions were enriched in the low-risk group (Figure 5(b)). For the CGGA-693 dataset, the collagen-activated signaling pathway and Hippo signaling were significantly enriched in the high-risk group (Figure 5(c)). Limited collagens expression was observed in the normal brain, while elevated collagen levels have been proven to drive glioma progression, which was in accordance with our results [30]. In addition, catabolic-related processes were highly enriched in the CGGA-693 low-risk group (Figure 5(d)). Similar functional results were found between the CGGA-325 and TCGA-LGG datasets (Figures 5(e) and 5(f)), which implies the robustness of the risk score in distinguishing high- and low-risk LGG patients.

### 3.6. Comparison of the TIME and Immune Signatures for the High- and Low-Risk Groups

**Figure 6:**
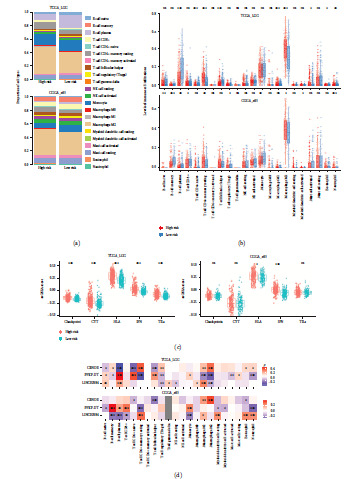
The TIME pattern and immune signatures of LGG risk groups. (a) Bar plots showing the proportion of immune cells in each LGG risk group for TCGA-LGG and CGGA-693 datasets. (b) Box plots showing the levels of 22 immune cell infiltrations between high- and low-risk groups for TCGA-LGG and CGGA-693 datasets. (c) The differences of immune signature activities estimated by ssGSEA between LGG risk groups. (d) The correlation between lncRNA expression and immune cell infiltrations.  ^*∗*^*p* < 0.05,  ^*∗∗*^*p* < 0.01,  ^*∗∗∗*^*p* < 0.001, and ns for no significant.

Since the GSEA results showed significant enrichment of immune regulatory function, we next investigated whether the constitution of the TIME and activities of immune signatures were different in the high- and low-risk groups. The immune landscapes of LGG patients for 22 immune cell types were first depicted via CIBERSORT (Figure 6(a) and Figure S7(A)). In general, there were relatively high proportions of M2 macrophages and monocytes in the LGG TIME. In the TCGA dataset, the infiltration levels of memory resting CD4+ T cells, M1 macrophages, M2 macrophages, activated mast cells, and neutrophils were increased in the high-risk group. The level of plasma B cells, naïve CD4+ T cells, follicular helper T cells, and eosinophils were significantly decreased (Figure 6(b)) in the high-risk group. For the CGGA-693 dataset, higher levels of naïve B cell, memory resting CD4+ T cells, M2 macrophages, activated myeloid dendritic cells, and neutrophils were observed, while lower levels of memory B cells, plasma B cells, CD8+ T cells, regulatory T cells (Tregs), and monocytes were observed in the high-risk group (Figure 6(b)). Although no significant results were found in the CGGA-325 cohort, similar trends between risk groups were observed, such as the increased level of M2 macrophages (Figure S7(B)). We next investigated the differences in immune-related signatures and found that the activities of all signatures were elevated in the high-risk group (Figure 6(c) and Figure S7(C)). The activities of immune inhibitors were significantly higher than those of immune stimulators in the high-risk group for all LGG datasets, which may be associated with the worse survival of these patients (Figure S8). Consistent with the prognostic coefficient from the Cox model, the expression of PFKP-DT was positively correlated with the levels of cancer-killing immune cells such as CD8+ T cells, while the expression levels of CRNDE and LINC02984 were positively related to the infiltration levels of M2 macrophage, whose predominance contributed to the suppression of immunity (Figure 6(d) and Figure S7(D)) [31].

### 3.7. Identification of Potential Compounds Ideal for Treating the Risk Groups

**Figure 7:**
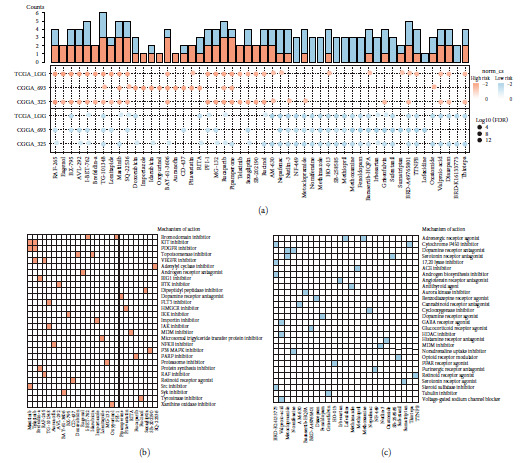
Explorations of compounds target the risk groups by CMap analysis. (a) Heatmap showing enrichment score of top 10 compounds for each LGG risk group. Compounds targeting high-risk groups were colored by red and low-risk groups in blue. (b, c) Heatmap showing each compound that shares the mechanism of actions (rows) in high- and low-risk groups. Sorted by descending number of the compound with a shared mechanism of actions.

To explore the candidate compounds ideal for treating m6A-related risk groups, CMap analysis was employed based on the top 150 differentially expressed genes between risk groups. The 10 most applicable compounds in the high- and low-risk groups for each LGG dataset are shown in Figure 7(a). Among them, several compounds, such as eugenol and brefeldin-a, were found to be specifically useful for patients in high-risk groups. We also identified that NF-449, nomifensine, methimazole, and other drugs were suitable for low-risk patients. MoA analysis revealed that bromodomain, KIT, PDGFR, topoisomerase, and VEGFR inhibitors were common targets of compounds suitable for the high-risk group (Figure 7(b)). For the low-risk group, adrenergic receptor agonists, cytochrome P450 inhibitors, dopamine receptor antagonists, and serotonin receptor antagonists were common targets of the different compounds (Figure 7(c)). Further studies are needed to verify the therapeutic value of these compounds for LGG patients.

## 4. Discussion

Tumor stemness has been attributed to postsurgery recurrence and therapeutic resistance in glioma patients [32]. Growing evidence has already shown that specific lncRNAs modified by m6A can influence the malignancy of tumors by regulating stemness in the glioma TIME [11, 33]. To determine the value of m6A-related lncRNAs in predicting LGG prognosis and stemness status, 1,247 LGG patients from multicenter datasets were enrolled in this study. The stem cell landscapes of LGG patients were characterized, and associations with clinical features were estimated. Consistent with previous findings [27], methylated MGMT, 1p/19q codeletion, and IDH mutation patients all possessed a higher mRNAsi. For WHO grades, patients with grade II exhibited increased mRNAsi scores in both the TCGA-LGG and CGGA-325 cohorts. A previous study reported that the negative correlation between mRNAsi and tumor pathology was restricted to LGG, which may be due to the reduced cell differentiation caused by IDH1 mutation [6, 34].

Previous studies have revealed the essential roles of m6A-related lncRNAs in tumor stemness [35]. Through integrating differentially expressed lncRNAs and their correlations with m6A regulators, we identified 364 robust m6A-related lncRNAs for LGG patients. Some lncRNAs have been reported to be modified by m6A and associated with tumors. For instance, YTHDF3 serves as an m6A reader to negatively regulate GAS5, thus triggering YAP phosphorylation and degradation and inhibiting the progression of colorectal cancer [36]. The m6A reader IGF2BP2 could regulate DANCR to promote cancer stemness-like properties and pathogenesis [37]. Moreover, DANCR has been reported to be strongly associated with glioma malignancy [38]. We constructed a risk signature based on the expression of LINC02984, PFKP-DT, and CRNDE and successfully sectionalized LGG patients into risk groups with different survival statuses. CRNDE is a well-known target for glioma treatment, and its great prognostic value has been proven at the bulk and single-cell levels in a previous study [39]. PFKP, the nearest protein-coding gene for PFKP-DT, has been reported to interact with VDAC2, thus regulating phenotypic reprograming and glucose metabolism of glioma stem cells [40]. Collectively, these findings highlight the critical roles of m6A-related lncRNAs in LGG stemness regulation and survival prediction.

The effect of tumor stemness on the TIME has recently been supported by experimental evidence [41]. We estimated the TIME of LGG tumors and found that patients in different risk groups exhibited heterogeneity for specific immune cells. The infiltration levels of memory resting CD4+ T cells, M2 macrophages, and neutrophils were all increased in high-risk groups across LGG datasets. As reported, macrophages are the most abundant cell types in the TIME for glioma, and the tumor-associated macrophages and their secreted factors are essential to the progression of glioma. Among them, M2 macrophages were proven to mediate protumor effects and lead to the suppression of systemic immunity [31, 42]. Moreover, evidence has demonstrated that neutrophils can support the expansion of the glioma stem cell pool via an S100 protein-dependent mechanism, thus promoting the glioblastoma progression, which was consistent with our study [43]. We also found that the plasma B cells, whose expansion can be a predictor of favorable patient survival [44], were decreased in high-risk groups. In addition, a decrease in CD8+ T cell infiltration and elevated activities of immune inhibitors were observed in the high-risk group. These results may partially explain the poor survival of the LGG patients in the high-risk groups.

We next made efforts to identify compounds that may be appropriate for the treatment of patients at different risk levels. Eugenol (a bioactive constituent present in essential oils) was found to specifically target factors enriched in high-risk patients. Li et al. [45] found that eugenol could induce apoptotic and antimetastatic activity through the MMP signaling pathway in a glioma rat model. Treatment with brefeldin-a significantly inhibited stem cell self-renewal and improved the survival of a glioma mouse model [46]. For low-risk groups, several drugs that have been reported were also identified. For instance, methimazole has been employed to treat glioblastoma in phase 2 clinical trials (accession numbers: NCT05607407 and NCT02654041). Moreover, the functions of methoxamine, TTNPB, and other compounds in inhibiting glioma cells have been previously described [47, 48]. Collectively, we indeed found highly promising compounds that may be applicable for glioma treatment.

## 5. Conclusion

In conclusion, we systemically estimated the association between tumor stemness and LGG clinical features. A risk model based on important m6A-related lncRNAs was constructed to predict LGG prognosis and stemness status was constructed. The differences in the TIME were explored, and potential compounds were identified between risk groups. The signature that we present will provide novel insight into clinical regimens for LGG patients.

## Data Availability

The public transcriptome profiles and clinical data were provided in the Materials and Methods section. Software and resources used for the analyses are described in each Materials and Methods section.

## Supplementary Materials

Supplementary Materials Figure S1: violin plots of mRNAsi in TCGA LGG patients classified by (A) ATRX and (B) TERT mutation. Figure S2: gender and age distributions between mRNAsi-high and mRNAsi low groups in TCGA-LGG, CGGA-693, and CGGA-325 datasets. Figure S3: the least absolute shrinkage and selection operator Cox (LASSO-Cox) analysis for m6A-related lncRNAs. Figure S4: gender and age distributions between high-risk and low-risk groups in TCGA-LGG, CGGA-693, and CGGA-325 datasets. Figure S5: the performance of risk model in (A) GSE108471 and (B) E-MATB-3892 cohorts. Figure S6: the differences in stemness scores between high- and low-risk groups of LGG patients. Figure S7: the TIME pattern and immune signatures of CGGA-325 risk groups. Figure S8: the differences of activities for immune stimulators and inhibitors in high-risk and low-risk groups from TCGA-LGG, CGGA-693, and CGGA-325 cohorts. Table S1: the characteristics of datasets in this study. Table S2: stemness gene sets used in this study.
