# Supplementary material for: Development of an m6A-Related lncRNAs Signature Predicts Tumor Stemness and Prognosis for Low-Grade Glioma Patients
Source: Stem Cells Int. 2024 Jan 9;2024:2062283. doi: 10.1155/2024/2062283 (PMC10791469; doi:10.1155/2024/2062283)
Supplement: Supplementary Materials — Figure S1: violin plots of mRNAsi in TCGA LGG patients classified by (A) ATRX and (B) TERT mutation. Figure S2: gender and age distributions between mRNAsi-high and mRNAsi low groups in TCGA-LGG, CGGA-693, and CGGA-325 datasets. Figure S3: the least absolute shrinkage and selection operator Cox (LASSO-Cox) analysis for m6A-related lncRNAs. Figure S4: gender and age distributions between high-risk and low-risk groups in TCGA-LGG, CGGA-693, and CGGA-325 datasets. Figure S5: the performance of risk model in (A) GSE108471 and (B) E-MATB-3892 cohorts. Figure S6: the differences in stemness scores between high- and low-risk groups of LGG patients. Figure S7: the TIME pattern and immune signatures of CGGA-325 risk groups. Figure S8: the differences of activities for immune stimulators and inhibitors in high-risk and low-risk groups from TCGA-LGG, CGGA-693, and CGGA-325 cohorts. Table S1: the characteristics of datasets in this study. Table S2: stemness gene sets used in this study. [file 2062283.f1.pdf]

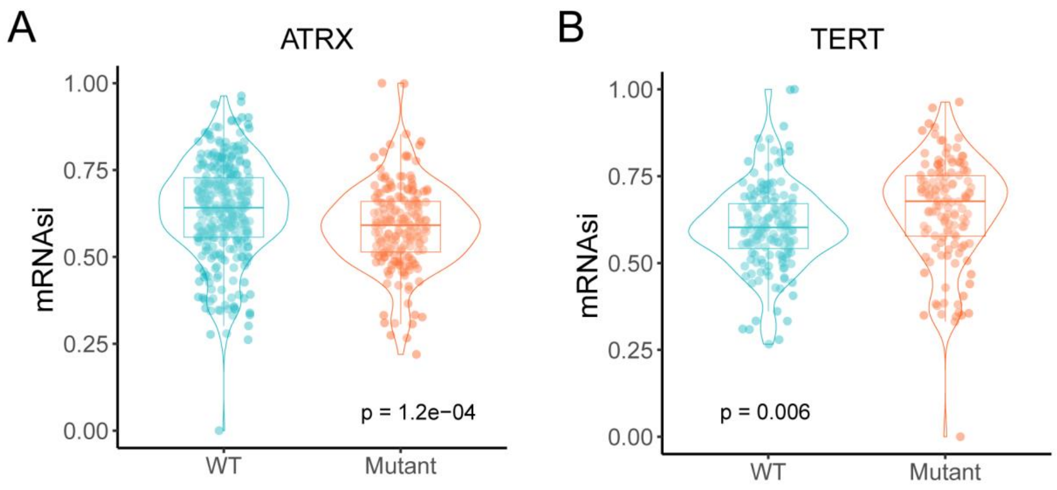

Figure S1. Violin plots of mRNAsi in TCGA LGG patients classified by (A) ATRX and (B) TERT mutation. Differential analysis between the LGG groups were estimated by the Student's t-test.

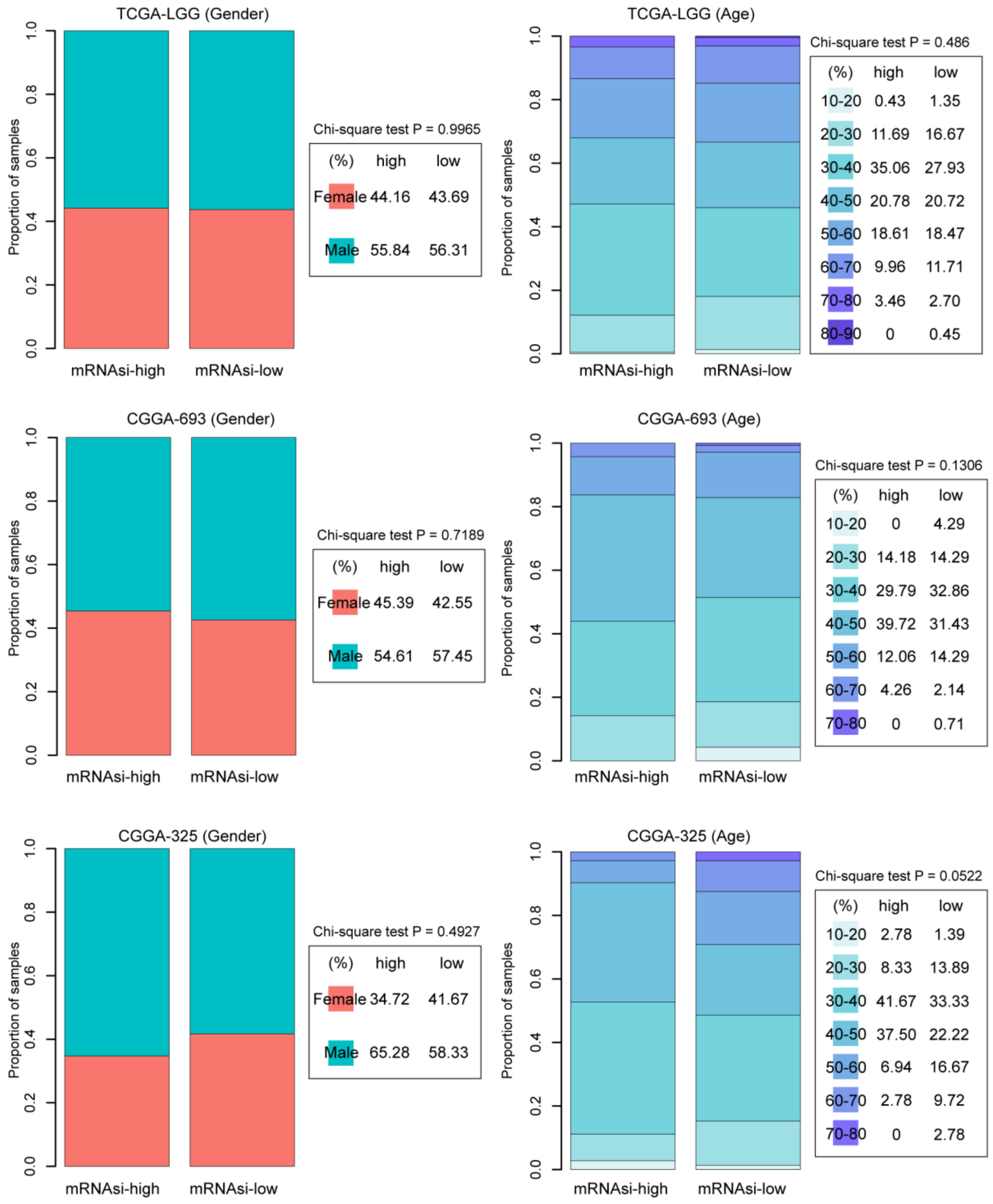

Figure S2. Gender and age distributions between mRNAsi-high and mRNAsi low groups in TCGA-LGG, CGGA-693, and CGGA-325 datasets.

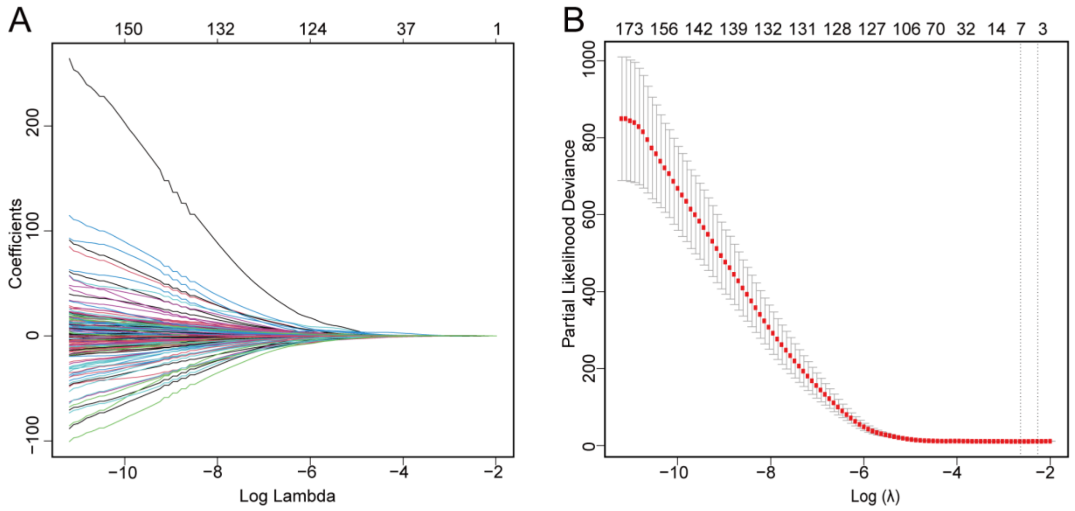

Figure S3. The least absolute shrinkage and selection operator Cox (LASSO-Cox) analysis for m6A-related lncRNAs. (A) The coefficients for m6A-related lncRNAs. (B) The best parameter (λ) was selected based on the LASSO model.

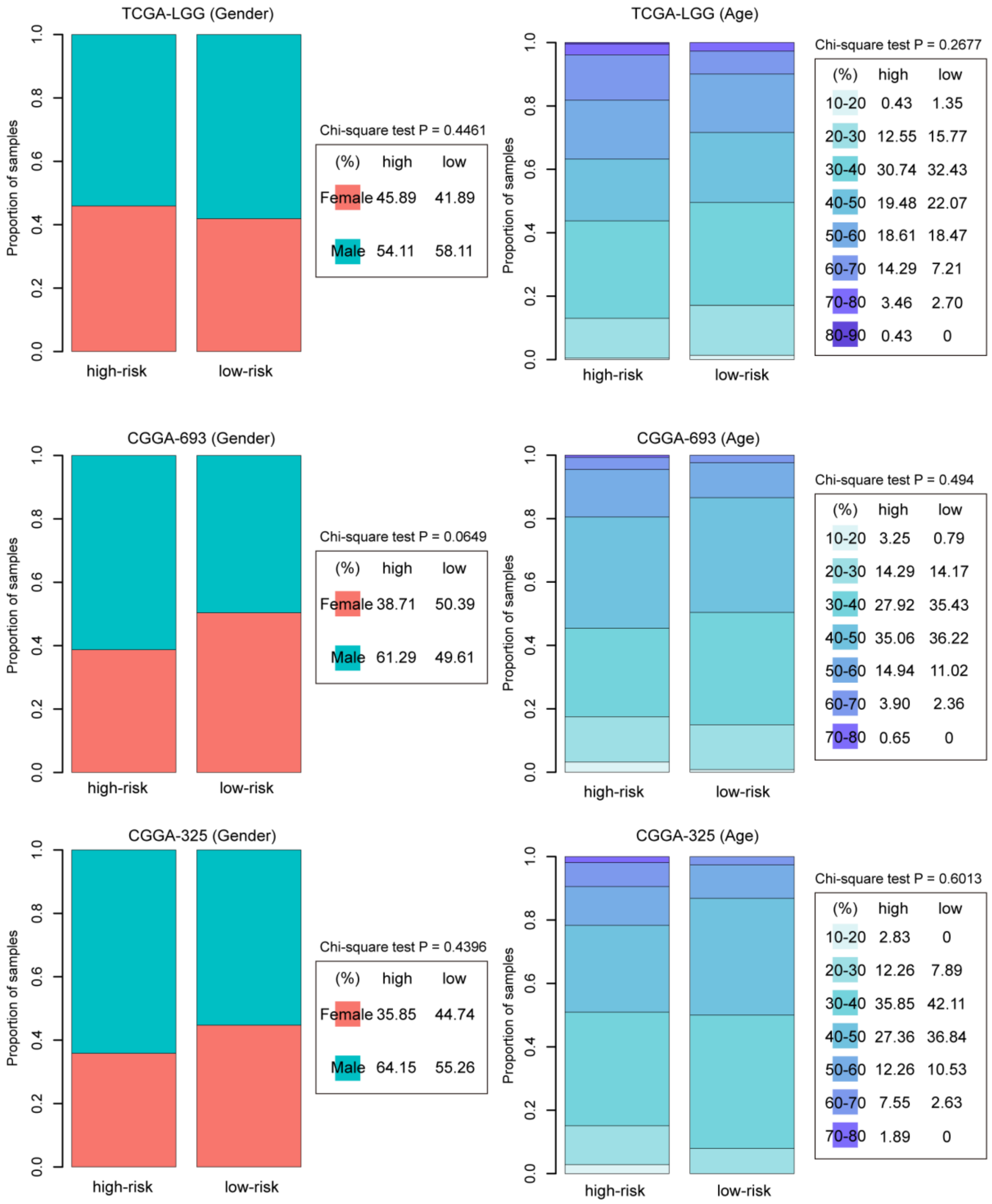

Figure S4. Gender and age distributions between high-risk and low-risk groups in TCGA-LGG, CGGA-693, and CGGA-325 datasets.

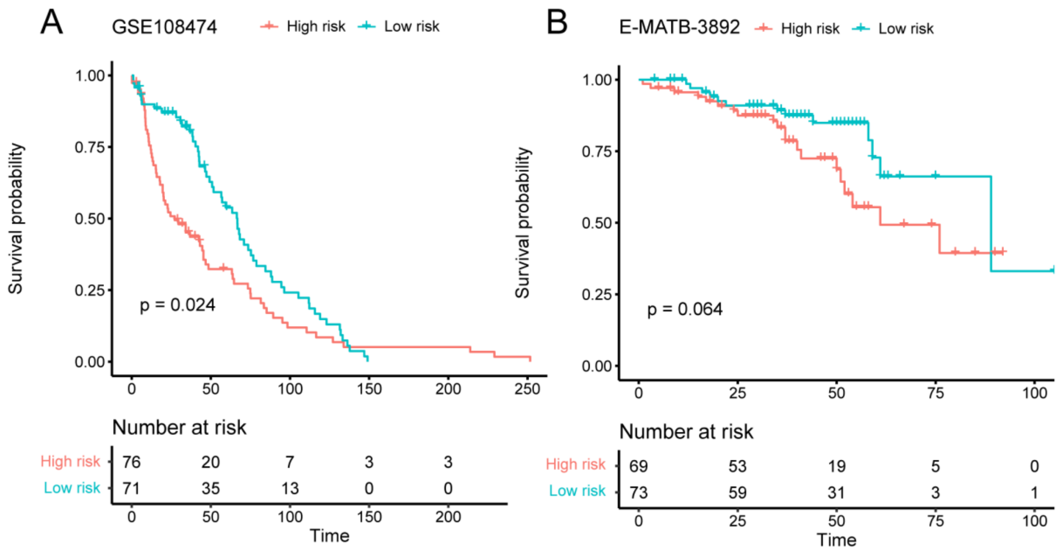

Figure S5. The performance of risk model in (A) GSE108471 and (B) E-MATB-3892 cohorts.

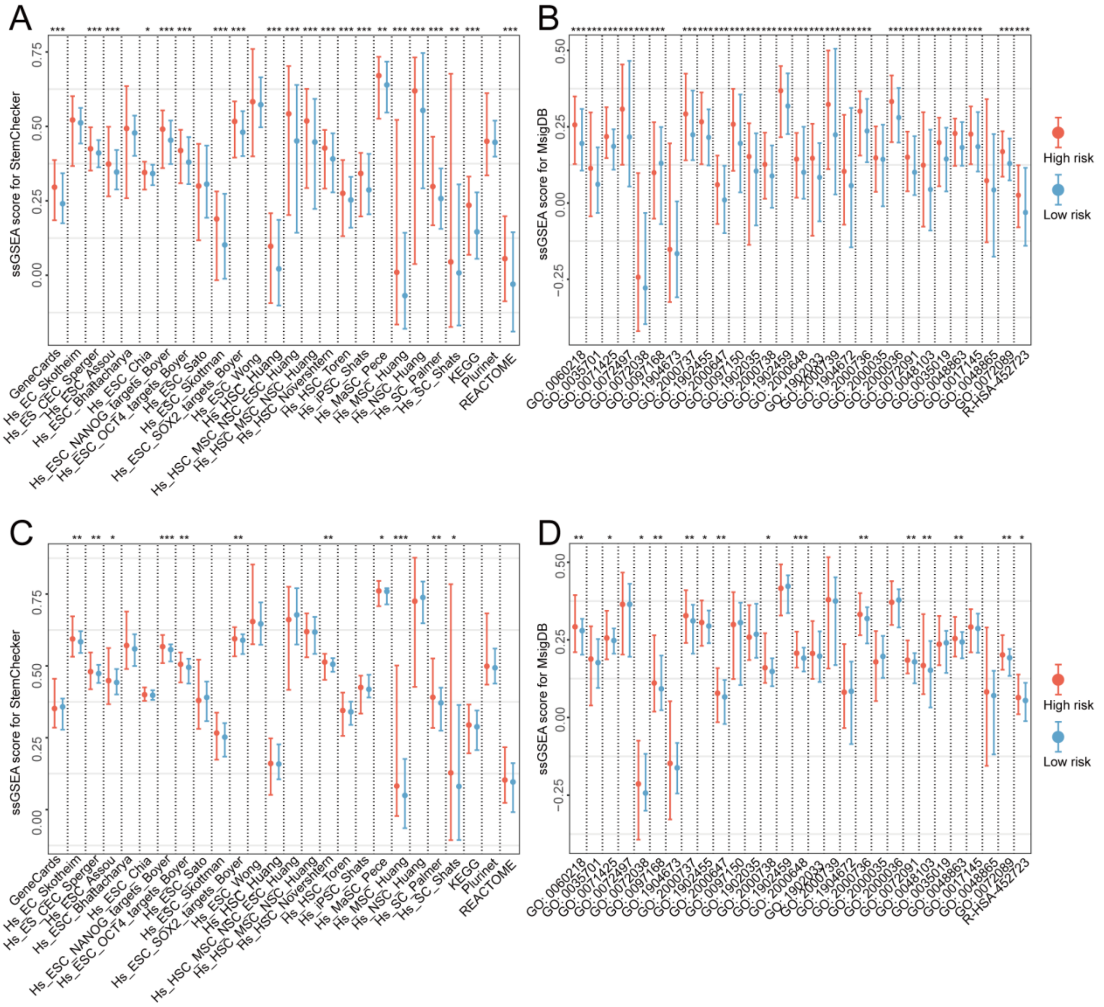

Figure S6. The differences in stemness scores between high- and low-risk groups of LGG patients. (A) and (B) Box plots showing the activities of StemChecker and MsigDB stemness categories estimated by ssGSEA in CGGA-693 risk groups. (C) and (D) Box plots showing the activities of StemChecker and MsigDB stemness categories estimated by ssGSEA in CGGA-325 risk groups. *: p < 0.05, **: p < 0.01, ***: p < 0.001 0.001, and ns for no significant.

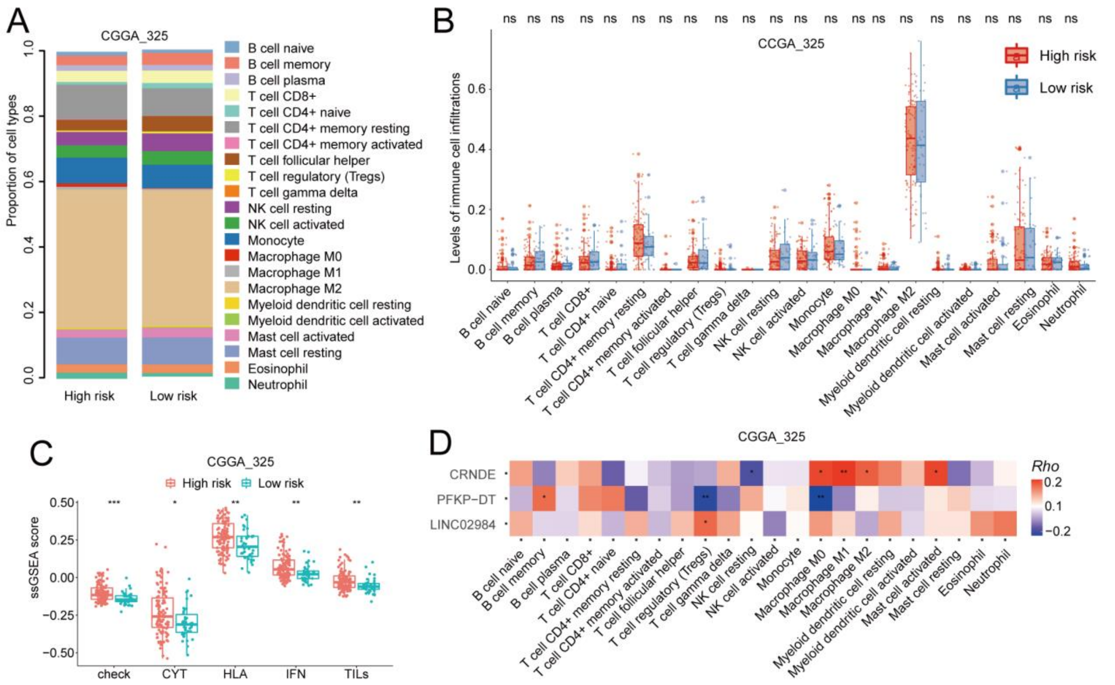

Figure S7. The TIME pattern and immune signatures of CGGA-325 risk groups. (A) Bar plots showing the proportion of immune cells in each LGG risk group. (B) Box plots showing the levels of 22 immune cell infiltrations between high- and low-risk groups. (C) The differences of immune signature activities estimated by ssGSEA between LGG risk groups. (D) The correlation between lncRNA expression and immune cell infiltrations. *: $p < 0.05$, **: $p < 0.01$, ***: $p < 0.001$ 0.001, and ns for no significant.

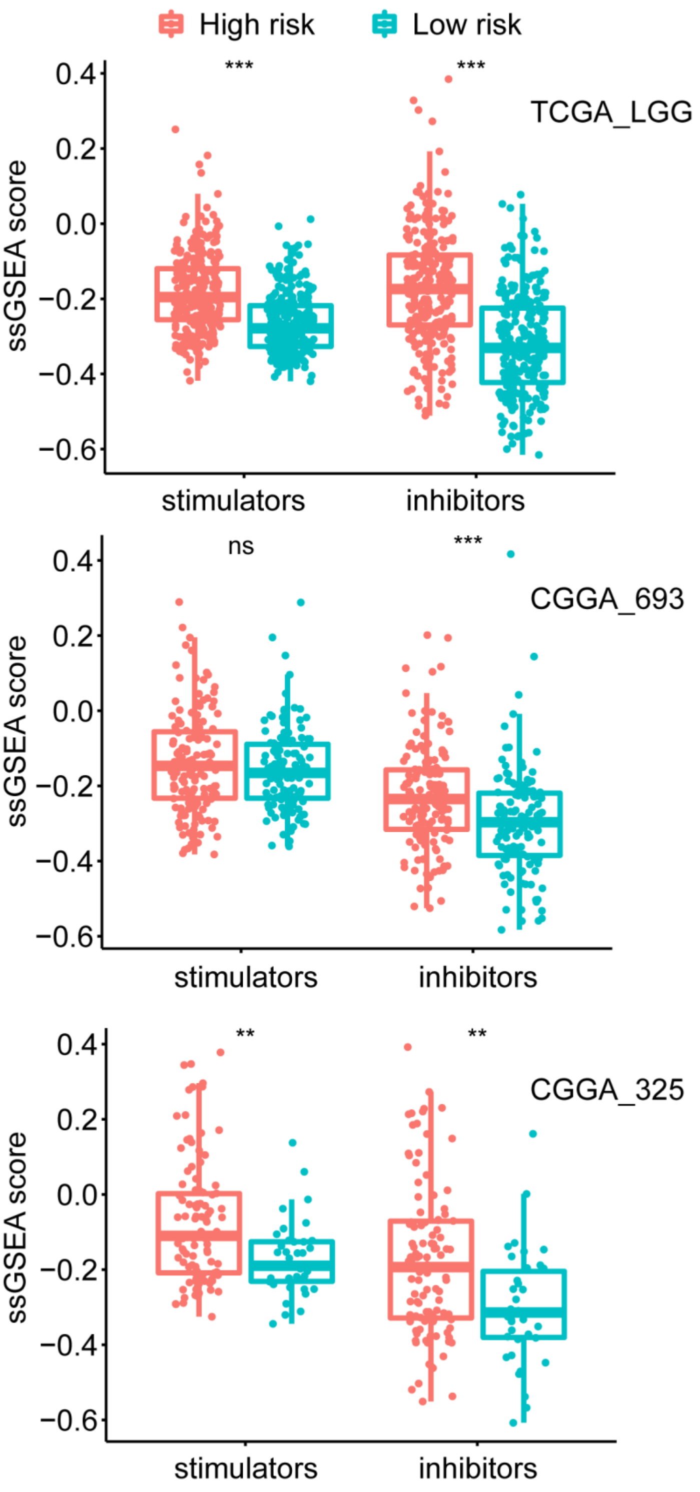

Figure S8. The differences of activities for immune stimulators and inhibitors in high-risk and low-risk groups from TCGA-LGG, CGGA-693, and CGGA-325 cohorts.

Table S1. The characteristic of datasets in this study.

Datasets	Sources	Platform	Samples
TCGA-LGG	TCGA	RNA-sequencing	511
CGGA-693	CGGA	RNA-sequencing	282
CGGA-325	CGGA	RNA-sequencing	144
GSE108474	GEO	microarray	162
E-MATB-3892	ArrayExpress	microarray	148

Table S2. Stemness gene sets used in this study.

Source	Catalogue	Numer of genes
StemChecker	GeneCards	133
StemChecker	Hs_EC_Skotheim	152
StemChecker	Hs_ES_CEC_Sperger	653
StemChecker	Hs_ESC_Assou	1066
StemChecker	Hs_ESC_Bhattacharya	89
StemChecker	Hs_ESC_Chia	507
StemChecker	Hs_ESC_NANOG_targets_Boyer	984
StemChecker	Hs_ESC_OCT4_targets_Boyer	289
StemChecker	Hs_ESC_Sato	17
StemChecker	Hs_ESC_Skottman	58
StemChecker	Hs_ESC_SOX2_targets_Boyer	731
StemChecker	Hs_ESC_Wong	335
StemChecker	Hs_HSC_Huang	94
StemChecker	Hs_HSC_MSC_NSC_ESC_Huang	18
StemChecker	Hs_HSC_MSC_NSC_Huang	114
StemChecker	Hs_HSC_Novershtern	854
StemChecker	Hs_HSC_Toren	106
StemChecker	Hs_iPSC_Shats	80
StemChecker	Hs_MaSC_Pece	306
StemChecker	Hs_MSC_Huang	59
StemChecker	Hs_NSC_Huang	54
StemChecker	Hs_SC_Palmer	184
StemChecker	Hs_SC_Shats	81
StemChecker	KEGG	136
StemChecker	Plurinet	299
StemChecker	REACTOME	28
MsigDB	GO:0060218; GOBP_HEMATOPOIETIC_STEM_CELL_DIFFERENTIATION	35
MsigDB	GO: 0035701; GOBP_HEMATOPOIETIC_STEM_CELL_MIGRATION	9
MsigDB	GO: 0071425; GOBP_HEMATOPOIETIC_STEM_CELL_PROLIFERATION	35
MsigDB	GO: 0072497; GOBP_MESENCHYMAL_STEM_CELL_DIFFERENTIATION	12
MsigDB	GO: 0072038; GOBP_MESENCHYMAL_STEM_CELL_MAINTENANCE_INVOLVED_IN_NEPHRON_MORPHOGENESIS	6
MsigDB	GO: 0097168; GOBP_MESENCHYMAL_STEM_CELL_PROLIFERATION	8
MsigDB	GO: 1904673; GOBP_NEGATIVE_REGULATION_OF_SOMATIC_STEM_CELL_	5

	POPULATION_MAINTENANCE	
MsigDB	GO: 2000737 ; GOBP_NEGATIVE_REGULATION_OF_STEM_CELL_DIFFERENTIATION	27
MsigDB	GO: 1902455; GOBP_NEGATIVE_REGULATION_OF_STEM_CELL_POPULATION_MAINTENANCE	26
MsigDB	GO: 2000647; GOBP_NEGATIVE_REGULATION_OF_STEM_CELL_PROLIFERATION	27
MsigDB	GO: 0097150; GOBP_NEURONAL_STEM_CELL_POPULATION_MAINTENANCE	23
MsigDB	GO: 1902035; GOBP_POSITIVE_REGULATION_OF_HEMATOPOIETIC_STEM_CELL_PROLIFERATION	9
MsigDB	GO: 2000738; GOBP_POSITIVE_REGULATION_OF_STEM_CELL_DIFFERENTIATION	24
MsigDB	GO: 1902459; GOBP_POSITIVE_REGULATION_OF_STEM_CELL_POPULATION_MAINTENANCE	47
MsigDB	GO: 2000648; GOBP_POSITIVE_REGULATION_OF_STEM_CELL_PROLIFERATION	55
MsigDB	GO: 1902033 ; GOBP_REGULATION_OF_HEMATOPOIETIC_STEM_CELL_PROLIFERATION	16
MsigDB	GO: 2000739; GOBP_REGULATION_OF_MESENCHYMAL_STEM_CELL_DIFFERENTIATION	8
MsigDB	GO: 1904672; GOBP_REGULATION_OF_SOMATIC_STEM_CELL_POPULATION_MAINTENANCE	11
MsigDB	GO: 2000736; GOBP_REGULATION_OF_STEM_CELL_DIFFERENTIATION	83
MsigDB	GO: 2000035; GOBP_REGULATION_OF_STEM_CELL_DIVISION	12
MsigDB	GO: 2000036; GOBP_REGULATION_OF_STEM_CELL_POPULATION_MAINTENANCE	71
MsigDB	GO: 0072091; GOBP_REGULATION_OF_STEM_CELL_PROLIFERATION	92
MsigDB	GO: 0048103; GOBP_SOMATIC_STEM_CELL_DIVISION	12

MsigDB	GO: 0035019; GOBP_SOMATIC_STEM_CELL_POPULATION_MAINTENANCE	67
MsigDB	GO: 0048863; GOBP_STEM_CELL_DIFFERENTIATION	249
MsigDB	GO: 0017145; GOBP_STEM_CELL_DIVISION	31
MsigDB	GO: 0048865; GOBP_STEM_CELL_FATE_COMMITMENT	10
MsigDB	GO: 0072089; GOBP_STEM_CELL_PROLIFERATION	124
MsigDB	R-HSA-452723; REACTOME_TRANSCRIPTIONAL_REGULATION_OF_PLURIPOTENT_STEM_CELLS	31
